# Transient Nutrient Deprivation Promotes Macropinocytosis-Dependent Intracellular Bacterial Community Development

**DOI:** 10.1128/mSphere.00286-18

**Published:** 2018-09-12

**Authors:** Rachael L. Hardison, Derek R. Heimlich, Alistair Harrison, Wandy L. Beatty, Sarah Rains, M. Arthur Moseley, J. Will Thompson, Sheryl S. Justice, Kevin M. Mason

**Affiliations:** a Center for Microbial Pathogenesis, The Research Institute at Nationwide Children’s Hospital, Columbus, Ohio, USA; b The Ohio State University College of Medicine, Columbus, Ohio, USA; c Department of Molecular Microbiology, Washington University School of Medicine, St. Louis, Missouri, USA; d Duke Proteomics and Metabolomics Core Facility, Duke Center for Genomic and Computational Biology, Duke University, Durham, North Carolina, USA; e Department of Pediatrics, The Ohio State University, Columbus, Ohio, USA; University of Kentucky

**Keywords:** *Haemophilus*, NTHI, endolysosomal pathway, host cell invasion, intracellular bacterial community, macropinocytosis, nutritional immunity

## Abstract

Nutrient limitation restricts bacterial growth in privileged sites such as the middle ear. Transient heme-iron restriction of nontypeable Haemophilus influenzae (NTHI), the major causative agent of chronic and recurrent otitis media (OM), promotes new and diverse phenotypes that can influence planktonic, biofilm, and intracellular lifestyles of NTHI. However, the bacterial responses to nutrient restriction that impact intracellular fate and survival of NTHI are unknown. In this work, we provide evidence for the role of transient heme-iron restriction in promoting the formation of intracellular bacterial communities (IBCs) of NTHI both *in vitro* and *in vivo* in a preclinical model of OM. We show that transient heme-iron restriction of NTHI results in significantly increased invasion and intracellular populations that escape or evade the endolysosomal pathway for increased intracellular survival. In contrast, NTHI continuously exposed to heme-iron traffics through the endolysosomal pathway for degradation. The use of pharmacological inhibitors revealed that prior heme-iron status does not appear to influence NTHI internalization through endocytic pathways. However, inhibition of macropinocytosis altered the intracellular fate of transiently restricted NTHI for degradation in the endolysosomal pathway. Furthermore, prevention of macropinocytosis significantly reduced the number of IBCs in cultured middle ear epithelial cells, providing evidence for the feasibility of this approach to reduce OM persistence. These results reveal that microenvironmental cues can influence the intracellular fate of NTHI, leading to new mechanisms for survival during disease progression.

**IMPORTANCE** Otitis media is the most common bacterial infection in childhood. Current therapies are limited in the prevention of chronic or recurrent otitis media which leads to increased antibiotic exposure and represents a significant socioeconomic burden. In this study, we delineate the effect of nutritional limitation on the intracellular trafficking pathways used by nontypeable Haemophilus influenzae (NTHI). Moreover, transient limitation of heme-iron led to the development of intracellular bacterial communities that are known to contribute to persistence and recurrence in other diseases. New approaches for therapeutic interventions that reduce the production of intracellular bacterial communities and promote trafficking through the endolysosomal pathway were revealed through the use of pharmacological inhibition of macropinocytosis. This work demonstrates the importance of an intracellular niche for NTHI and provides new approaches for intervention for acute, chronic, and recurring episodes of otitis media.

## Abstract (precis)

Otitis media is the most common bacterial infection in childhood. Current therapies are limited in the prevention of chronic or recurrent otitis media which leads to increased antibiotic exposure and represents a significant socioeconomic burden. In this study, we delineate the effect of nutritional limitation on the intracellular trafficking pathways used by nontypeable Haemophilus influenzae (NTHI). Moreover, transient limitation of heme-iron led to the development of intracellular bacterial communities that are known to contribute to persistence and recurrence in other diseases. New approaches for therapeutic interventions that reduce the production of intracellular bacterial communities and promote trafficking through the endolysosomal pathway were revealed through the use of pharmacological inhibition of macropinocytosis. This work demonstrates the importance of an intracellular niche for NTHI and provides new approaches for intervention for acute, chronic, and recurring episodes of otitis media.

## INTRODUCTION

Bacterial pathogens exploit diverse host niches to survive and cause disease. Opportunistic pathogens, by definition, are especially adaptable as the host microenvironment can shift rapidly during the transition from a commensal to a pathogen. Physiological stressors occur as a result of innate and adaptive host immune defense mechanisms and can drive phenotypic changes that promote diverse bacterial lifestyles. Understanding the mechanisms underlying responses to microenvironmental cues that influence bacterial survival in both extracellular (planktonic and biofilm) and intracellular (vacuolar and cytosolic) environments is critical to ultimately prevent chronic and/or recurrent diseases.

As an opportunistic pathogen, nontypeable Haemophilus influenzae (NTHI) is a major cause of otitis media (OM), exacerbations of chronic obstructive pulmonary disease, and sinusitis, among others (1–4). Many NTHI infections can return despite prior antibiotic treatment, and NTHI is the most commonly isolated microbe from recurrent episodes of OM (5–11).

The survival and pathogenicity of NTHI, a heme-iron auxotroph, depend on the ability to acquire iron from the external environment. NTHI resides as a commensal in the nasopharynx but can migrate into the sterile middle ear under permissive conditions such as an upper respiratory viral infection or Eustachian tube dysfunction (12, 13). At the onset of OM, the host tightly sequesters heme-iron and other essential nutrients through nutritional immunity. Over the course of infection, inflammation and immune responses cause fluctuations in iron availability. NTHI has multiple mechanisms for iron acquisition and responds to these changes by upregulating core iron- and heme-responsive genes during experimental OM (14). Additionally, heme acquisition genes were more prevalent in NTHI strains isolated from the middle ears of children with OM than in strains isolated from the throats of healthy children (15). These studies underscore the importance for NTHI heme-iron acquisition in development of disease.

Iron limitation can induce phenotypic changes that promote virulence and survival in bacteria, including stimulation of biofilm formation in Staphylococcus aureus and increased adherence of Pseudomonas aeruginosa to epithelial cells (16, 17). We have recently demonstrated that transient heme-iron restriction of NTHI alters biofilm architecture and morphology, increases survival in a preclinical model of OM, and promotes invasion and intracellular bacterial community (IBC) formation within chinchilla middle ear epithelial (CMEE) cells (18–20).

While historically considered an extracellular pathogen, intracellular NTHI is observed within respiratory and middle ear epithelia (7, 21, 22). NTHI is internalized by host cells through cell-type-specific mechanisms that include clathrin-mediated endocytosis, actin remodeling, microtubules, and lipid rafts (23–30). Further, multiple studies have demonstrated that NTHI may enter cells through macropinocytosis (28, 31). Once internalized, NTHI typically traffics via the endolysosomal pathway, resulting in degradation within lysosomes (22, 23). However, the contribution of intracellular NTHI to the disease progression of recurrent and chronic OM and, importantly, how nutritional immunity and resulting heme-iron restriction may influence the intracellular fate of NTHI remain underexplored.

Our prior studies have revealed a potential viable intracellular population of NTHI in response to heme-iron limitation (18, 20). Based upon these observations, we hypothesized that prior heme-iron restriction of NTHI alters intracellular bacterial trafficking and results in increased bacterial survival within host cells. Here, we report that heme-iron restriction of NTHI leads to productive invasion into epithelial cells *in vitro* and *in vivo* that promotes IBC formation in chinchilla middle ear epithelium during experimental OM. In contrast to NTHI that was continuously exposed to heme-iron, we observed populations of transiently restricted NTHI that did not colocalize with markers of the early and late endolysosomal pathway, suggesting evasion or escape from early endocytic vacuoles. Further, our data demonstrate that entry via macropinocytosis contributes to intracellular survival and evasion of endolysosomes by transiently restricted NTHI. Together, these data reveal a novel role for the response of NTHI to changes in heme-iron availability in promoting an intracellular lifestyle and advance our understanding of how NTHI may survive within the host middle ear epithelium to cause acute and recurrent OM.

## RESULTS

### Transient heme-iron restriction of NTHI promotes the formation of intracellular bacterial communities in an experimental model of OM.

**FIG 1:**
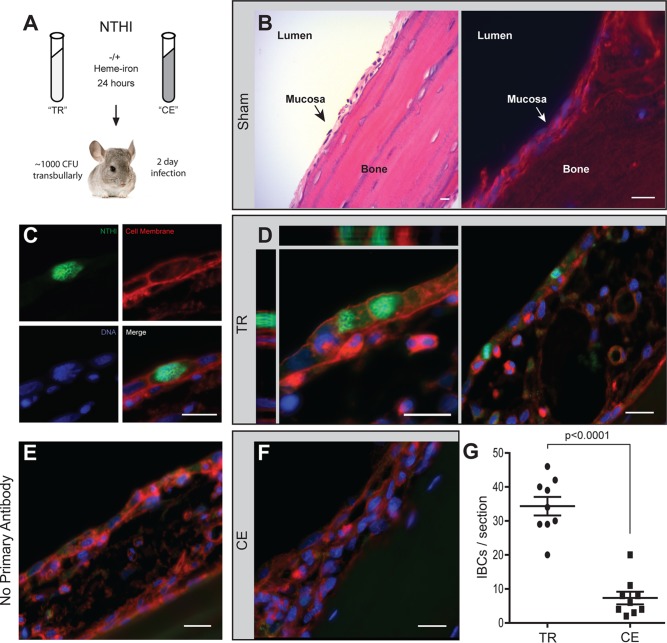
Transient heme-iron restriction of NTHI promotes intracellular bacterial community formation in a preclinical model of otitis media. (A) Schematic representation of environmental heme-iron conditioning of NTHI strain 86-028NP and inoculation of chinchilla middle ears. Two days postinoculation, middle ears were excised, fixed, and paraffin embedded and thin sections from sham-treated, transiently restricted, or continuously exposed infected ears were processed for microscopy. TR, transiently restricted NTHI; CE, continuously exposed NTHI. (B) Four-nanometer thin sections of sham-treated middle ear bullae were stained with hematoxylin and eosin (left panel) or processed for immunofluorescence microscopy and stained with wheat germ agglutinin and 4′,6-diamidino-2-phenylindole (right panel). The lumen, mucosa, and bone are denoted for anatomical orientation. (C) Immunofluorescence micrograph gallery depicting the surface staining of transiently restricted NTHI by anti-OMP labeling (green), staining of the host cell membrane by wheat germ agglutinin (red), and staining of host and bacterial DNA by Hoechst stain (blue). (D) Representative images of transiently restricted NTHI IBCs within middle ear mucosal epithelium, including a three-dimensional rendering of a series of optical sections (left panel) to depict the orthogonal views of IBCs filling the entirety of the epithelial cell and visualized using the fluorophores depicted in panel C. Multiple IBCs were observed in each thin section. (E) A no-primary-antibody control of a thin section of a middle ear infected with transiently restricted NTHI. The thin section is sequential to those in panel D and depicts the specificity of NTHI labeling. (F) Representative image of middle ears infected with continuously exposed NTHI depicts the absence of IBCs in these thin sections. Bar, 10 µm for all images. (G) Quantification of IBCs in thin sections of ears infected with transiently restricted or continuously exposed NTHI. Three sequential thin sections were counted for three ears from each cohort. Significance was determined using a two-tailed Student *t* test.

Heme-iron restriction of NTHI alters bacterial interactions with host epithelial cells. In our prior studies using strains transiently depleted of heme-iron, we observed an increase in the populations of intracellular NTHI within cultured epithelial cells (18, 20). Although NTHI can internalize into epithelial cells, the fate is typically nonproductive due to trafficking into degradative pathways (22, 23). In contrast, when NTHI is transiently restricted for heme-iron, we observe large intracellular clusters of bacteria reminiscent of IBCs growing within cultured middle ear epithelial cells (20). Within clinical biopsy specimens, intracellular bacteria have been observed in middle ear epithelium obtained from children with OM (32). Therefore, we sought to determine whether prior heme-iron restriction of NTHI would promote the formation of IBCs by NTHI within the chinchilla middle ear mucosal epithelium in a preclinical model of OM. The prototypical NTHI strain 86-028NP was cultured in defined iron source (DIS) medium in the presence or absence of 2 µg/ml heme-iron for 24 h, resulting in two cultures, one continuously exposed to heme-iron (CE) and one transiently restricted of heme-iron (TR) (Fig. 1A). The cultures were normalized for viable counts and inoculated directly into the chinchilla middle ear (Fig. 1A). Two days postinoculation, middle ears were processed to evaluate the formation of IBCs within the middle ear mucosae (Fig. 1A). The anatomy of the middle ear includes epithelial mucosae typically consisting of 2 to 3 layers of cells overlaying a bony septum (Fig. 1B). NTHI was visualized within thin sections using an antiserum directed against NTHI outer membrane proteins (OMPs) (green, Fig. 1C). In addition, counterstains were included to visualize the host cell membranes (red) and DNA (blue) (Fig. 1C). We first observed that infection with either transiently restricted or continuously exposed NTHI (Fig. 1D and F) resulted in the expansion of the middle ear epithelium compared with the sham-treated ear (Fig. 1B). Infection with transiently restricted NTHI resulted in the formation of IBCs that filled the volume of the cells, and multiple IBCs were observed within each thin section (Fig. 1D). The staining of IBCs using the antiserum was specific for NTHI as evidenced by the absence of fluorescence in the green channel when the primary antibody was excluded (Fig. 1E). In contrast to the transiently restricted NTHI, intracellular clusters of NTHI were not readily observed within the mucosal layer of chinchilla middle ears infected with continuously exposed NTHI (Fig. 1F). Although we did observe instances of intracellular populations of continuously exposed NTHI, the number of IBCs per thin section was significantly lower in ears infected with continuously exposed NTHI compared to transiently restricted NTHI (Fig. 1G). Continuously exposed NTHI resided mainly in a biofilm with immune cell infiltrate localized on top of the epithelial mucosae. Hence, as observed in other highly recurrent infections (33), we observe a productive intracellular lifestyle for NTHI during experimental OM as a consequence of nutritional status.

### Heme-iron restriction significantly increases intracellular survival of NTHI.

**FIG 2:**
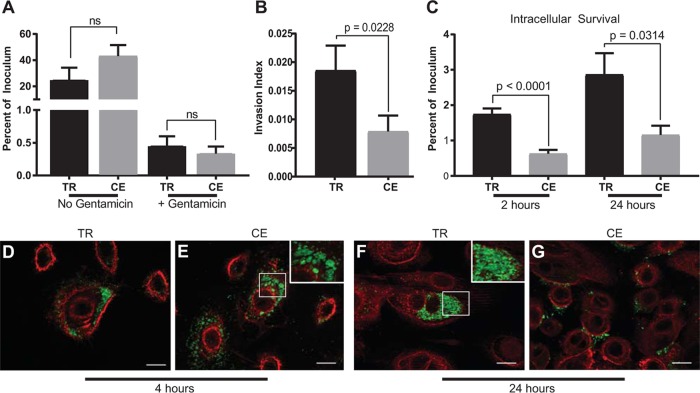
Transiently restricted NTHI invades and survives within human epithelial cells in intracellular bacterial communities. (A) Normal human bronchial epithelial (NHBE) cells were cocultured with either transiently restricted (TR) or continuously exposed (CE) NTHI for 1 h, and total bacterial association (no gentamicin) or intracellular bacteria only (+ gentamicin) were quantified. Results are depicted as the percentage of inoculum that remained viable with or without gentamicin treatment. Statistical significance was determined using a two-tailed Student *t* test of the means from duplicate wells from each of three biological replicates. Error bars indicate standard errors of the means. ns, not significant. (B) The invasion index of transiently restricted (TR) and continuously exposed (CE) NTHI defined as the number of viable intracellular bacteria when normalized for total association. Statistical significance was determined using a two-tailed Student *t* test on the mean from technical duplicates from each of three biological replicates. Error bars represent standard errors of the means. (C) Intracellular survival of transiently restricted (TR) and continuously exposed (CE) NTHI following infection of NHBE cells for 2 or 24 h. Results are depicted as the percentage of inoculum that remained viable following gentamicin treatment. Statistical significance was determined using a two-tailed Student *t* test on the mean from duplicates from each of eight biological replicates for the 2-h time point and five biological replicates for the 24-h time point. Error bars represent standard errors of the means. (D to G) NTHI strain 86-028NP(pGM1.1) expressing green fluorescent protein was transiently restricted (TR) or continuously exposed (CE) to heme-iron and then cocultured with NHBE cells for 4 (D and E) or 24 (F and G) hours. Epithelial cell membranes were labeled with wheat germ agglutinin conjugated to Alexa Fluor 594 (red), and bacteria were visualized by GFP fluorescence (green). Bar, 10 µm. Images depict the formation of early IBCs by TR NTHI as early as 4 h postinoculation (D) that progress to mature IBCs at 24 h (F). CE NTHI localizes in circular compartments (E, inset) and does not form IBCs 24 h postinoculation (G).

The differences in the formation of IBCs between the transiently restricted and continuously exposed NTHI suggest that nutritional conditioning increases the association with and survival within middle ear epithelial cells. Technical limitations prohibit quantification of intracellular bacteria at early time points *in vivo*. We therefore capitalized upon our *in vitro* culture model to evaluate the ability of NTHI to survive within primary normal human bronchial epithelial (NHBE) cells (18, 20). Gentamicin protection assays were used to distinguish between NTHI association (adherent and invaded NTHI) and invasion (protected intracellular NTHI) following transient heme-iron restriction. There was no significant difference in either association or the initial invasion of transiently restricted or continuously exposed NTHI with NHBE cells (Fig. 2A). However, when invasion was normalized for the total number of bacteria associated with the cells, we observed a statistically significant increase in the intracellular populations of the transiently restricted NTHI compared with the continuously exposed NTHI (Fig. 2B). Consistent with our *in vivo* and *in vitro* observations (Fig. 1C and D and Fig. 2B), there was a statistically significant increase in the intracellular survival of transiently restricted NTHI following gentamicin treatment for at least 24 h (Fig. 2C). Neither the continuously exposed nor transiently restricted NTHI was cytotoxic to NHBE cells at any time point (see Fig. S1 in the supplemental material). Although there was a trend for an increase in the association of the continuously exposed NTHI compared with the transiently restricted NTHI (Fig. 2A), there was no significant difference in the percentage of intracellular bacteria upon titration of the multiplicity of infection (MOI) (Fig. S2). These observations suggest that there are differences in the subcellular localization of the transiently restricted and continuously exposed NTHI. We would predict that the transiently restricted NTHI population occupies niches that allow productive growth whereas continuously exposed NTHI proceeds through a degradative pathway. Using fluorescent reporter strains, we observed that both transiently restricted and continuously exposed NTHI were internalized into NHBE monolayers at 4 h (Fig. 2D and E). For the transiently restricted NTHI, we observed intracellular bacteria that resemble the early stages of IBC formation (Fig. 2D). In contrast, the continuously exposed NTHI appeared to be compressed into circular structures, suggesting that the bacteria are within membrane-enclosed compartments (Fig. 2E). The intracellular location of NTHI is supported by the use of specific antisera in the presence and absence of permeabilization of host cell membranes to identify intracellular and extracellular bacteria, respectively (Fig. S3). Consistent with our previously published reports using primary respiratory epithelial cells (18, 20), we observed increased intracellular populations of transiently restricted NTHI within the cytoplasm of NHBE cells at 24 h postinoculation (Fig. 2F), while intracellular populations of continuously exposed NTHI decreased and did not progress to IBC formation (Fig. 2G). Therefore, we provide evidence using gentamicin protection, specific antibody labeling, and optical sectioning approaches that transiently restricted and continuously exposed NTHI reside in different subcellular locations. Taken together, our data demonstrate that transient heme-iron restriction of NTHI promotes formation of IBCs within epithelial cells.

10.1128/mSphere.00286-18.2 FIG S1 Nutritionally conditioned NTHI is not cytotoxic to NHBE cells. The potential cytotoxicity of transiently restricted (TR) or continuously exposed (CE) NTHI to NHBE cells at 4 or 24 h was assessed by the release of lactate dehydrogenase (LDH). Experiments were performed in biological duplicate, and data are reported as the fold change in cytotoxicity, compared to uninfected cells. Error bars represent standard errors of the means. The maximum release of LDH was determined by the lysis of all cells with 9% Triton X-100. Neither TR nor CE NTHI displayed a cytotoxic effect on NHBE cells at any time point used in this study. Download FIG S1, EPS file, 0.4 MB. Copyright © 2018 Hardison et al. 2018 Hardison et al. This content is distributed under the terms of the Creative Commons Attribution 4.0 International license.

10.1128/mSphere.00286-18.3 FIG S2 Effect of multiplicity of infection on intracellular survival of NTHI. Intracellular survival assays at 2 h postinoculation were performed with NHBE cells inoculated with transiently restricted (TR) or continuously exposed (CE) bacteria at a multiplicity of infection (MOI) of 1, 12.5, 25, 50, or 100 per cell. The percentage of viable inoculum remaining following gentamicin protection is reported. Data represent the means from duplicate wells for each of three biological replicates with standard errors of the means. Download FIG S2, EPS file, 0.4 MB. Copyright © 2018 Hardison et al. 2018 Hardison et al. This content is distributed under the terms of the Creative Commons Attribution 4.0 International license.

10.1128/mSphere.00286-18.4 FIG S3 Intracellular localization of nutritionally conditioned NTHI in the presence and absence of permeabilization. To confirm the intracellular localization of transiently restricted (TR) or continuously exposed (CE) NTHI in NHBE cells, confluent monolayers were cocultured with NTHI for 4 hours. Cells were permeabilized or nonpermeabilized in parallel and analyzed by immunofluorescence. Epithelial cell membranes were visualized by wheat germ agglutinin conjugated to Alexa Fluor 594 (red), and DNA (bacterial and host) was visualized by 4′,6-diamidino-2-phenylindole (DAPI) and pseudocolored white. NTHI was labeled with anti-OMP and detected with protein A-Alexa Fluor 488 (green). In nonpermeabilized cells (left), intracellular bacteria are white (indicated by white arrows) and extracellular bacteria are green/white (indicated by green arrows). TR NTHI is frequently observed within cells (top left panel) while CE NTHI is mostly external with few bacteria residing intracellularly. In permeabilized cells (right), all bacteria regardless of nutritional condition or localization are visualized as green/white. Bar, 10 µm. Download FIG S3, TIF file, 2.3 MB. Copyright © 2018 Hardison et al. 2018 Hardison et al. This content is distributed under the terms of the Creative Commons Attribution 4.0 International license.

### Heme-iron restriction of NTHI alters bacterial trafficking to early endosomes.

**FIG 3:**
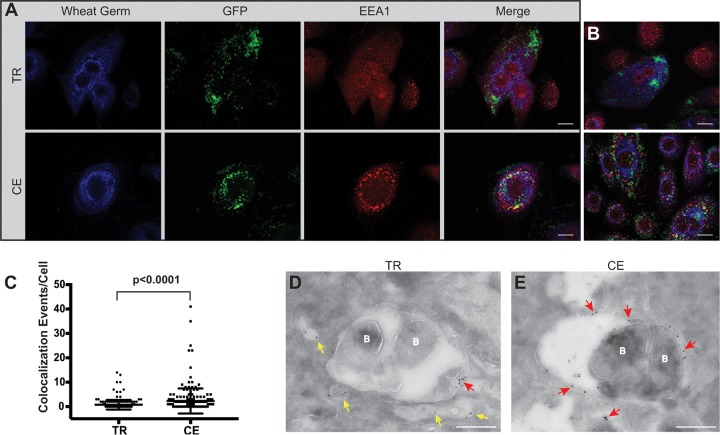
Transient heme-iron conditioning of NTHI alters trafficking to early endosomes. (A) NHBE cells were cocultured with either transiently restricted (TR) or continuously exposed (CE) NTHI strain 86-028NP(pGM1.1) for 4 h. Epithelial cell membranes were labeled with wheat germ agglutinin conjugated to Alexa Fluor 350 (blue), and bacteria were visualized by GFP fluorescence (green). Early endosomes were labeled with rabbit antibody to early endosomal antigen 1 (EEA1) protein and visualized with donkey anti-rabbit IgG conjugated to Alexa Fluor 594 (red). Representative images are shown for each condition (TR or CE) with individual and merged fluorescence images shown for depiction of colocalization. Colocalization of bacteria with EEA1 is observed as either yellow (merged) or red EEA1 label closely surrounding clusters of green NTHI bacteria. Bar, 10 µm. (B) Additional images representative of those depicted in panel A. (C) The number of colocalization events per cell was quantified by visual assessment of 200 individually infected cells. Statistical significance was determined by Mann-Whitney U test, and error bars represent standard errors of the means. (D and E) Transmission electron microscopy of NHBE cells cocultured with TR or CE conditioned NTHI for 4 h and subsequently immunolabeled to detect bacterial association with EEA1. Early endosomes were labeled with rabbit antibody to EEA1 and visualized with anti-rabbit IgG antibody conjugated to an 18-nm colloidal gold particle. EEA1-containing vesicles devoid of bacteria (labeled B) are indicated by yellow arrows, while red arrows indicate EEA1-containing vesicles associated with bacteria. Bar, 500 nm.

The ability of NTHI to grow within the cell following transient restriction of heme-iron, combined with the differential subcellular localization of the continuously exposed NTHI, suggests that nutritional conditioning promotes NTHI escape or evasion of vacuolar trafficking to gain access to the cytoplasm. Consistent with published observations, once internalized into epithelial cells, continuously exposed NTHI appears to remain contained within circular compressed compartments (Fig. 2E). Thus, we would predict that the continuously exposed NTHI is trafficked through the endolysosomal pathway (22, 23). The observation of significant increases in intracellular survival of NTHI following prior heme-iron restriction of the bacteria (Fig. 2C) led us to hypothesize that continuously exposed NTHI would be more often associated with markers of the endolysosomal pathway than the transiently restricted NTHI. To evaluate the differential trafficking of the transiently restricted and continuously exposed NTHI, NHBE cells were cocultured with NTHI and subsequently examined for bacterial colocalization with the specific marker of early endosomes, early endosomal antigen 1 (EEA1) (34). We observed continuously exposed NTHI within EEA1-containing membrane compartments at 4 h postinfection (Fig. 3A, B, and E). The EEA1 signal was distributed around the periphery of the entire compartment (Fig. 3E, red arrows). Colocalization of continuously exposed NTHI within EEA1-containing membranes was observed throughout the NHBE cells. In contrast, transiently restricted NTHI did not appear to be localized within EEA1-containing compartments and appeared free in the cytoplasm (Fig. 3A and B). In addition, EEA1 appeared to be associated with smaller vesicles that do not contain NTHI (Fig. 3D, yellow arrows). In some cases, we did observe EEA1 in close proximity to the transiently restricted NTHI, but the protein was not distributed peripherally around the entire membrane compartment (Fig. 3D, red arrow). The number of NTHI and EEA1 colocalization events was quantitatively assessed from 200 independent cells infected with either transiently restricted or continuously exposed NTHI and visualized by fluorescence microscopy (Fig. 3C). The observation of a significant association of continuously exposed NTHI within EEA1 membrane-bound compartments is consistent with prior observations (22, 23). However, the transiently restricted NTHI demonstrates a different intracellular localization pattern with significantly fewer colocalization events per cell (Fig. 3C). Based upon these observations, we predict that transiently restricted NTHI would not enter into the later stages of the lysosomal pathway and promote intracellular survival.

### Infection with transiently restricted NTHI changes LAMP1 distribution.

**FIG 4:**
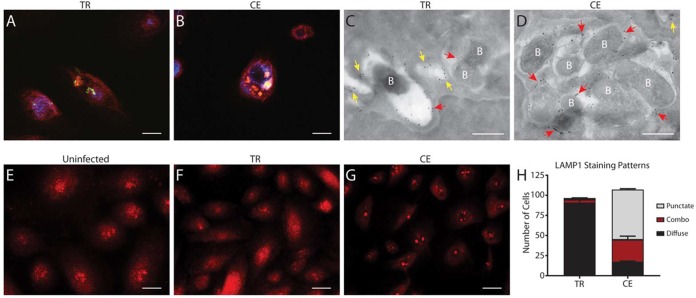
Transient heme-iron restriction of NTHI alters lysosomal trafficking and biogenesis. (A and B) NHBE cells were cocultured with transiently restricted (TR) or continuously exposed (CE) NTHI strain 86-028NP(pGM1.1) and assessed for colocalization of NTHI with LAMP1 by immunofluorescence at 24 h postinoculation. Cell membranes were visualized with wheat germ agglutinin conjugated to Alexa Fluor 350 (blue), and NTHI was visualized by GFP fluorescence (green). LAMP1 bar, 10 µm. (C and D) Colocalization of TR and CE NTHI with LAMP1 was assessed by TEM. LAMP1 was labeled with rabbit anti-LAMP1 and detected with anti-rabbit IgG conjugated to 18-nm colloidal gold. LAMP1-positive vesicles devoid of bacteria (labeled B) are indicated by yellow arrows, and LAMP1-positive vesicles associated with bacteria are indicated with red arrows. Bar, 500 nm. (E to G) LAMP1 staining patterns depict differences in lysosomal biogenesis in NHBE cells infected with TR NTHI (F) or CE NTHI (G) at 5 h postinoculation. Uninfected cells are included for comparison (E). LAMP1 was visualized using rat anti-LAMP1 and detected with anti-rat IgG conjugated to Alexa Fluor 594 (red). TR NTHI infection promotes a diffuse LAMP1 staining pattern while CE NTHI infection promotes a punctate and often perinuclear staining pattern in NHBE cells. Uninfected NHBE cells display a combination of both diffuse and punctate staining. Bar, 10 µm. (H) LAMP1 staining patterns were quantified by visual counting of 100 individual NHBE cells from each condition (infected with either TR or CE NTHI). Statistical significance was determined using a two-tailed Student *t* test.

Endosomes function to sort and direct internal cargo for eventual fusion with lysosomes for targeted degradation (35). Given the strong association of continuously exposed NTHI with EEA1, we predict that the fate of the continuously exposed NTHI would involve continuation through the endolysosomal pathway. To test this, we investigated the colocalization of lysosomal associated membrane protein 1 (LAMP1) with transiently restricted or continuously exposed NTHI. At 24 h, transiently restricted NTHI did not colocalize with LAMP1, whereas there was evident colocalization of continuously exposed NTHI with LAMP1 (Fig. 4A and B). These data suggest that continuously exposed NTHI traffics to the lysosomes whereas transiently restricted NTHI avoids or escapes this pathway. Vacuole-associated LAMP1 was also observed by immunogold transmission electron microscopy (TEM) (Fig. 4C and D). As we observed with EEA1, LAMP1 was distributed around vacuolar compartments that contained continuously exposed NTHI (Fig. 4D, red arrows). In contrast, while we did observe transiently restricted NTHI associated with LAMP1 (Fig. 4C, red arrows), we more often observed LAMP1-containing vesicles devoid of NTHI (Fig. 4C, yellow arrows). Interestingly, we observed striking differences in the subcellular distribution of LAMP1 signal by immunofluorescence microscopy, dependent on whether NTHI was transiently restricted or continuously exposed (Fig. 4F and G). Uninfected NHBE cells displayed LAMP1 signal that consisted of a mixture of diffuse and punctate staining patterns (Fig. 4E). Infection of NHBE cells with continuously exposed NTHI (Fig. 4G) results in a punctate LAMP1 staining pattern that appears to be centrally located within the cell. In marked contrast, infection of NHBE cells with transiently restricted NTHI (Fig. 4F) results in a LAMP1 staining pattern that is almost entirely diffuse, suggesting that localization of LAMP1 is altered. Visual quantification of staining patterns per ∼100 cells confirmed that infection with transiently restricted NTHI promotes a diffuse LAMP1 distribution in close to 90% of cells while infection with continuously exposed NTHI promotes this phenotype in only 20% of cells, with the majority of cells displaying a staining pattern of punctate LAMP1 (Fig. 4H). Taken together, these data demonstrate that transiently restricted NTHI evades the lysosomes and may alter lysosomal biogenesis.

### Internalization of NTHI into epithelial cells is prevented with pharmacological inhibition of endocytosis.

**FIG 5:**
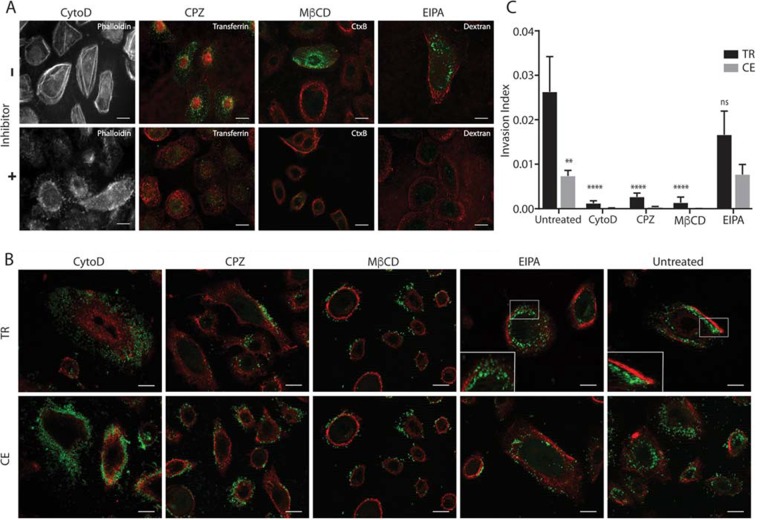
Pharmacological inhibition of endocytosis pathways reveals that nutritionally conditioned NTHI is internalized into cells through multiple mechanisms. (A) Representative images depicting inhibition of uptake of known fluorescent cargo conjugates (Alexa Fluor 488, green) into NHBE cells in the presence of each pharmacological inhibitor [CPZ, chlorpromazine; MβCD, methyl-β-cyclodextrin; EIPA, 5-(*N*-ethyl-*N*-isopropyl)-amiloride]. The optimal concentration of cytochalasin D (CytoD) was determined by staining F-actin with phalloidin conjugated to Alexa Fluor 350. Epithelial cell membranes were labeled with wheat germ agglutinin conjugated to Alexa Fluor 594 (red). Bar, 10 µm. (B) Fluorescence microscopy was used to determine uptake of transiently restricted (TR) or continuously exposed (CE) NTHI strain 86-028NP(pGM1.1) following pharmacological inhibition of endocytosis pathways. NHBE cells were pretreated with pharmacological inhibitors prior to a 4-h incubation with TR or CE NTHI strain 86-028NP(pGM1.1). NTHI was visualized by GFP fluorescence (green), and epithelial cell membranes were labeled with wheat germ agglutinin conjugated to Alexa Fluor 594 (red). The far-right panel depicts infected cells with no inhibitor (Untreated) for comparison. Each experiment was performed in three biological replicates, and representative images are shown. Bar, 10 µm. (C) Viable intracellular bacteria were enumerated following gentamicin treatment of NHBE cells that were incubated with transiently restricted (TR) or continuously exposed (CE) NTHI for 1 h in the presence of pharmacological inhibitors compared to untreated controls. Statistical significance was determined by analysis of variance with means from duplicate wells from three independent biological replicates, and error bars represent standard errors of the means (**, *P*  < 0.01; ****, *P*  < 0.0001). The invasion index was calculated as the number of viable intracellular bacteria divided by total associated bacteria.

The differential trafficking of transiently restricted and continuously exposed NTHI could be a consequence of the mechanism of internalization. Proteomic analysis indicates that the outer membrane profiles are similar between the transiently restricted and continuously exposed NTHI, suggesting that these two populations could use similar adhesins for initial interactions with the epithelial cells (Table 1). NTHI is internalized into the cell through multiple endocytic pathways (36). Therefore, we hypothesized that transiently restricted and continuously exposed NTHI may be internalized into epithelial cells through different pathways. To investigate this, NHBE monolayers were pretreated with a panel of pharmacological inhibitors to target endocytic pathways (Table S1) prior to coculture with transiently restricted or continuously exposed NTHI. The effective dosage for each inhibitor was empirically determined with the use of appropriate indicators of endocytosis (i.e. cargo or cellular ultrastructure) (Fig. 5A and Table S1). There was no significant difference in the association of NTHI with epithelial cells in the presence or absence of the inhibitors (Fig. S4). In the absence of inhibition (Fig. 5B, untreated), intracellular populations were observed for both the transiently restricted and continuously exposed NTHI, consistent with our prior observations (Fig. 2D and E). We observed inhibition of internalization for both transiently restricted and continuously exposed NTHI in the presence of cytochalasin D (CytoD; an inhibitor of actin polymerization), chlorpromazine (CPZ; an inhibitor of clathrin or receptor-mediated endocytosis), and methyl-β-cyclodextrin (MβCD; an inhibitor of caveolae/lipid raft domains) as evidenced by the presence of extracellular bacteria on the cell periphery (Fig. 5B). The extent of NTHI internalization in the presence and absence of the inhibitors was further quantified using gentamicin protection (Fig. 5C). We observed statistically significant decreases in internalization of transiently restricted NTHI upon addition of these three inhibitors, suggesting that internalization of transiently restricted NTHI can occur through endocytic pathways. Although internalization was significantly reduced, treatment with these three inhibitors did not completely abolish internalization of transiently restricted NTHI, suggesting an alternate route of entry. Although the initial invasive populations of continuously exposed NTHI were significantly reduced from the transiently restricted NTHI (Fig. 2B and 5C), treatment with CytoD, CPZ, and MβCD almost completely abolished internalization of continuously exposed NTHI (Fig. 5B and C).

**TABLE 1:** Assessment of differentially expressed proteins of NTHI transiently restricted and continuously exposed to heme-iron^*a*^

NCBI protein GI	NTHI no.	Gene	Annotation	Protein description	Peptide no.	Fold change	*P* value
GI:895836 505	NTHI0175		Conserved hypothetical	Class I SAM-dependent methyltransferase	6	6	0.0005
GI:915525 770	NTHI1895	*tehB*	Tellurite resistance protein	Tellurite resistance methyltransferase TehB	5	4.5	0.0034
GI:100606 0856	NTHI0351	*queA*	*S*-Adenosylmethionine:tRNA ribosyltransferase-isomerase	tRNA preQ1(34) *S*-adenosylmethionine ribosyltransferase-isomerase QueA	4	3.1	0.003
GI:499591 591	NTHI1169	*tbp2*	Transferrin-binding protein 2	Transferrin-binding protein-like solute binding protein	8	3	0.0075
GI:501001 680	NTHI0034	*lipB*	Lipoate-protein ligase B	Lipoyl(octanoyl) transferase LipB	3	2.5	0.0048
GI:764356 334	NTHI0605	*hisF*	Imidazole glycerol phosphate synthase subunit HisF	Imidazole glycerol phosphate synthase subunit HisF	3	1.9	0.009
GI:915492 423	NTHI1899		Conserved hypothetical protein	30S ribosomal protein S12 methylthiotransferase accessory protein YcaO	4	1.8	0.0031
GI:499591 843	NTHI1649	*fabA*	3-Hydroxydecanoyl-[ACP] dehydratase	Beta-hydroxydecanoyl-ACP dehydratase	9	1.6	0.0078
GI:915560 482	NTHI1099	*hktE*	Catalase	Catalase	2	−2.2	0.0098
GI:491953 725	NTHI1002	*frdA*	Fumarate reductase flavoprotein subunit	Fumarate reductase (quinol) flavoprotein subunit	15	−2.4	0.002
GI:499591 611	NTHI1207	*dmsA*	Anaerobic dimethyl sulfoxide reductase chain A precursor	Dimethyl sulfoxide reductase subunit A	9	−2.9	0.0002
GI:100603 3258	NTHI1000	*frdB*	Fumarate reductase iron-sulfur protein	Succinate dehydrogenase/fumarate reductase iron-sulfur subunit	6	−3.1	0.0045
LIPA_HA ES1*	NTHI0033	*lipA*	Lipoic acid synthetase	Lipoyl synthase OS = Haemophilus somnus	2	−3.2	0.0092
GI:100603 3084	NTHI1236	*cyoA*	Probable cytochrome oxidase subunit I	Cytochrome *d* terminal oxidase subunit 1	6	−4.2	0.0025
GI:116877 7694	NTHI1230	*nrfA*	Cytochrome *c*_552_	Ammonia-forming nitrite reductase cytochrome *c*_552_ subunit	11	−20.1	0.0027

a LC-MS/MS was performed on three independent biological replicates of 24-h planktonic cultures of transiently restricted (TR) or continuously exposed (CE) NTHI. A total of 6,973 peptides and 1,183 proteins were quantified. Proteins represented with a single peptide count were excluded. Of the remaining proteins, those which passed a fold change cutoff of ±1.5 and *P* < 0.01 are included in the table. Differential expression determination was based on calculation of the fold change (positive numbers indicate proteins that are more represented in the TR population; negative numbers indicate proteins that are more represented in the CE population), as determined by a two-tailed Student *t* test. ACP, acyl carrier protein.

10.1128/mSphere.00286-18.5 FIG S4 Association of nutritionally conditioned NTHI with NHBE cells is unchanged in the presence of pharmacological inhibitors. Total association (intracellular and extracellular) of transiently restricted (TR) or continuously exposed (CE) NTHI with NHBE cells pretreated with the indicated pharmacological inhibitors was determined at 1 h postinoculation. Data represent the means from duplicate wells from each of three biological replicates, and the standard error of the mean is shown. Addition of pharmacological inhibitors did not affect the total association of either TR or CE NTHI with NHBE cells. CytoD, cytochalasin D; CPZ, chlorpromazine; MβCD, methyl-β-cyclodextrin; EIPA, 5-(*N*-ethyl-*N*-isopropyl)-amiloride. Download FIG S4, EPS file, 0.4 MB. Copyright © 2018 Hardison et al. 2018 Hardison et al. This content is distributed under the terms of the Creative Commons Attribution 4.0 International license.

10.1128/mSphere.00286-18.7 TABLE S1 Pharmacological compounds used to inhibit endocytosis in this study. Download Table S1, PDF file, 0.2 MB. Copyright © 2018 Hardison et al. 2018 Hardison et al. This content is distributed under the terms of the Creative Commons Attribution 4.0 International license.

### Inhibition of macropinocytosis promotes trafficking of transiently restricted NTHI to early endosomes and decreases intracellular survival.

NTHI has been shown to use macropinocytosis for internalization into epithelial cells (28, 31). Since we observed uptake of both transiently restricted and continuously exposed NTHI through endocytic mechanisms (Fig. 5B and C), we next asked whether there was a difference in the uptake of these two NTHI populations by macropinocytosis. The effective dosage for 5-(*N*-ethyl-*N*-isopropyl)-amiloride (EIPA; an inhibitor of macropinocytosis) was empirically determined with the use of 70,000-molecular-weight (MW) dextran (Fig. 5A). Pretreatment of NHBE monolayers with EIPA had no effect on the internalization of the continuously exposed NTHI. In contrast, EIPA reduced but did not significantly inhibit internalization of transiently restricted NTHI compared to untreated cells (Fig. 5B and C). Thus, our data reveal a subpopulation of transiently restricted NTHI that is susceptible to inhibition of macropinocytosis that was not observed with the continuously exposed NTHI.

In addition to the subtle differences in the internalization of continuously exposed or transiently restricted NTHI in the presence of EIPA, we observed a striking difference in the intracellular localization of transiently restricted NTHI following uptake in EIPA-treated cells. Microscopic analysis revealed that in the presence of EIPA, transiently restricted NTHI now appeared to traffic similarly to the continuously exposed NTHI and was localized within circular structures, resembling vesicles or membrane compartments (Fig. 5B, inset). This was a remarkable difference from untreated cells, where transiently restricted NTHI appears free in the cytoplasm in early IBCs (Fig. 5B, untreated, and Fig. 2D). In contrast, EIPA did not alter the subcellular localization of the continuously exposed NTHI (Fig. 5B). Therefore, we hypothesized that EIPA promotes trafficking of transiently restricted NTHI through the endolysosomal pathway.

**FIG 6:**
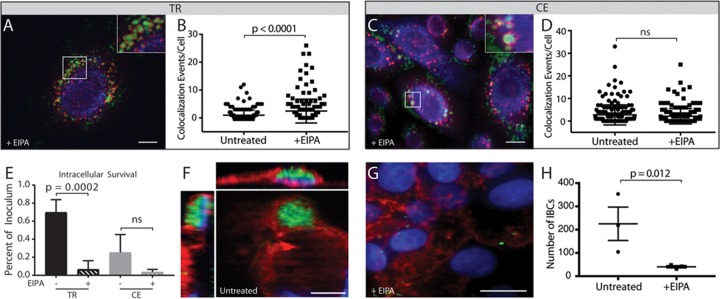
Inhibition of macropinocytosis redirects transiently restricted NTHI to the endolysosomal pathway and decreases intracellular survival. (A to D) NHBE cells were pretreated with 60 µM EIPA prior to coculture with transiently restricted (TR) or continuously exposed (CE) NTHI strain 86-028NP(pGM1.1) and visualized for colocalization of NTHI with EEA1 by fluorescence microscopy. NTHI was visualized by GFP fluorescence (green), EEA1 was labeled with rabbit antibody to EEA1 and visualized with donkey anti-rabbit IgG conjugated to Alexa Fluor 594 (red), and epithelial cell membranes were visualized with wheat germ agglutinin conjugated to Alexa Fluor 350 (blue). Representative images depict colocalization of TR (A) or CE (C) NTHI with EEA1 as observed by yellow fluorescence or red EEA1 labeling closely surrounding clusters of green NTHI bacteria. Bar, 10 µm. (B) EIPA pretreatment of NHBE cells significantly increases the number of colocalization events of TR NTHI with EEA1 in infected cells compared to infected cells that were not treated with EIPA. (D) Colocalization of CE NTHI with EEA1 does not significantly change in the presence or absence of EIPA. Statistical significance was determined by Mann-Whitney U test of colocalization events from a total of 200 independent cells from three biological assays. (E) Viable intracellular bacteria were enumerated following gentamicin treatment of NHBE cells infected with TR or CE NTHI in the presence and absence of EIPA. Statistical significance was determined by two-tailed Student’s *t* test of the means for duplicate wells from three independent biological replicates, and error bars represent standard errors of the means. (F to H) Intracellular bacterial communities were enumerated in chinchilla middle ear epithelial cells incubated with TR NTHI in the presence or absence of EIPA. (F and G) Bacteria were visualized by GFP fluorescence (green), epithelial cell members were stained with wheat germ agglutinin conjugated to Alex Fluor 594 (red), and host and bacterial DNA were labeled with Hoechst stain (blue). Bar, 10 µm. (H) Statistical significance was determined by two-tailed Student’s *t* test of the mean from duplicate wells from each of three independent biological replicates, and error bars represent standard errors of the means.

To determine the fate of transiently restricted NTHI in the presence of EIPA, pretreated NHBE cells were cocultured with transiently restricted or continuously exposed NTHI to visualize colocalization with EEA1. As expected, treatment with EIPA did not alter the trafficking of continuously exposed NTHI to early endosomes (Fig. 6C and D). Consistent with our hypothesis, we observed significantly increased colocalization of transiently restricted NTHI with EEA1 in the presence of EIPA as shown by quantitative assessment of fluorescent images (Fig. 6A and B). These data suggest that blocking macropinocytosis does not prevent bacterial entry but rather promotes trafficking of transiently restricted NTHI to the endocytosis pathway.

Based upon the observation that transiently restricted NTHI is diverted into the endolysosomal pathway upon treatment with EIPA, we predicted that inhibition of macropinocytosis would decrease the intracellular survival of transiently restricted NTHI. As previously observed (Fig. 2C), the intracellular survival of continuously exposed NTHI was decreased compared with the transiently restricted NTHI (Fig. 6E). EIPA treatment significantly reduced the intracellular survival of transiently restricted NTHI (Fig. 6E). In addition, EIPA also reduced the intracellular survival of the continuously exposed NTHI, although this was not significant (Fig. 6E). Taken together, these data suggest that entry through macropinocytosis is the primary pathway that promotes intracellular survival of transiently restricted NTHI.

To determine whether the long-term survival associated with productive growth leading to IBC formation is inhibited by EIPA, cultured primary chinchilla middle ear epithelial (CMEE) cells were cocultured with transiently restricted NTHI in the presence and absence of EIPA. Consistent with our observations using the preclinical model of OM (Fig. 1), IBCs were readily observed in cultured CMEE cells at 24 h (Fig. 6F). Pretreatment with EIPA significantly reduced the number of IBCs formed by transiently restricted NTHI (Fig. 6G and H). Taken together, these data suggest that entry via the macropinocytosis pathway promotes intracellular survival of transiently restricted NTHI, leading to productive IBC formation.

## DISCUSSION

**FIG 7:**
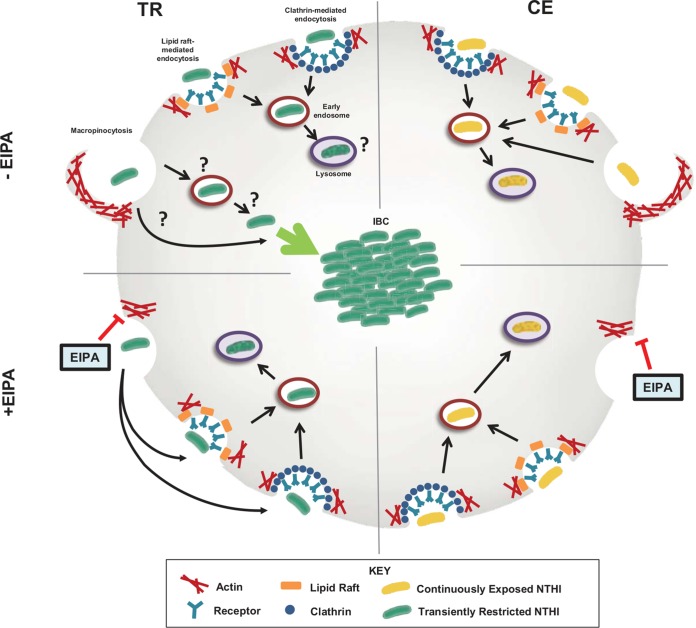
Proposed model for differential trafficking of TR NTHI through macropinocytosis resulting in IBC formation. In the absence of EIPA (top), both transiently restricted (TR, left) and continuously exposed (CE, right) NTHI enter the cells through endolysosomal pathways: clathrin-mediated endocytosis (blue circles), lipid raft/caveola-mediated endocytosis (orange rectangles), and macropinocytosis (membrane ruffling by actin polymerization, red). The ability to enter the cell through these various pathways appears to be independent of prior heme-iron status. Once internalized, CE NTHI (yellow bacteria) traffics to the early endosomes (red circles) and finally to the lysosomes (purple circles), where the bacteria are degraded. TR NTHI (green bacteria) also enters the cells through endolysosomal pathways and traffics to the early endosomes and lysosomes. In contrast, the subpopulation of transiently restricted NTHI that enters through macropinocytosis either completely evades or escapes this pathway (indicated by “?”) to form intracellular bacterial communities in the cell cytoplasm. In the presence of the macropinocytosis inhibitor EIPA (bottom), trafficking of continuously exposed NTHI through the endolysosomal pathway remains unchanged. Transiently restricted NTHI, entering the cell through clathrin- or lipid raft/caveola-mediated endocytosis, now localizes to the early endosomes. This shift in trafficking targets TR NTHI for degradation by the endolysosomal pathway and significantly decreases intracellular survival of this population.

Recent studies have revealed that NTHI can use multiple pathways for entry into host cells and typically traffics through the endolysosomal pathway for degradation within lysosomes (36). While we also observed that multiple mechanisms are used for NTHI internalization, our studies demonstrated that transient limitation of an essential nutrient redirects the fate of NTHI from a detrimental outcome to intracellular growth leading to IBCs (Fig. 1, 2, and 7). Pharmacological inhibition of various endocytic pathways demonstrated that the uptake of both transiently restricted and continuously exposed NTHI involves actin remodeling and can occur through clathrin- and lipid raft-dependent mechanisms (Fig. 5). Remarkably, inhibition of macropinocytosis ablated the formation of IBCs due to trafficking of NTHI through the endolysosomal pathway (Fig. 5 to 7). The formation of a viable intracellular population may provide a myriad of benefits, including protection from the host immune system, access to intracellular nutrients, or a respite from competing bacteria in polymicrobial infections. Additionally, intracellular reservoirs may be protected from antibacterial therapies that are the first line of treatment in many cases of OM (37). Taken together, nutrient availability is an important environmental cue for phenotypic responses culminating in an intracellular reservoir for NTHI.

Intracellular lifestyles are commonly associated with chronic and recurrent infections. IBCs were first described for uropathogenic Escherichia coli (UPEC) and were later found to act as reservoirs for recurrent infection (38–44). UPEC IBCs have been identified in bladder biopsy specimens and the urine of patients with urinary tract infections (45–48). More recently, the diversity of pathogens that form IBCs has expanded to include *Klebsiella*, *Proteus*, and *Helicobacter* (49–52). We now provide evidence for IBC formation in the pathogenesis of NTHI-mediated experimental OM (Fig. 1). Recent studies have demonstrated that UPEC IBCs remain metabolically active in the bladder (53), and intracellular populations of NTHI within endosomes were found to remain metabolically active for 24 h (22). Future studies will investigate the metabolic activity of NTHI IBCs and the contribution to persistence and reoccurrence of OM.

An important lingering question is the mechanism underlying the ability of transiently restricted NTHI to evade the endocytic pathway. Pharmacological inhibition suggests that the majority of NTHI, regardless of heme-iron status, is taken up through actin-mediated pathways. Furthermore, when either clathrin- or lipid raft-mediated endocytosis pathways were inhibited, we observed similar decreases in viable intracellular populations. Recent studies demonstrate that in addition to the removal of cholesterol to prevent lipid raft-mediated endocytosis, MβCD also impedes the formation of receptor-mediated clathrin-coated endocytic vesicles (54). Prior studies demonstrate that platelet activating factor receptor-mediated endocytosis predominates over macropinocytosis for uptake of NTHI (31). Thus, it appears that the subpopulation of transiently restricted NTHI which is taken up by macropinocytosis may be primed for increased survival within or escape from macropinosomes compared to the continuously exposed NTHI.

To determine the potential bacterial factors that contribute to differential trafficking and increased intracellular survival of NTHI, we compared the proteomes of transiently restricted and continuously exposed NTHI (Table 1). Although differential uptake and trafficking could result from differences in the composition of the outer membrane, our proteomics analysis did not reveal any differences in the profiles of adhesins and outer membrane proteins. We hypothesized that transient heme-iron restriction would change the NTHI protein profile that would promote survival in an intracellular niche. We identified 15 proteins that were significantly different in the TR population compared to the CE population (Table 1). As expected, a number of these proteins are members of the Fur regulon (55). Most of the proteins that were decreased in the TR population require iron-sulfur clusters for enzymatic activity (e.g., FrdAB, DmsA, and NrfA) (56–59). Further, NrfA, a subunit of a nitrite reductase, is a tetraheme cytochrome (59). Thus, downregulation of these proteins under heme-restricted conditions would preserve heme-iron stores.

Interesting roles may exist for the proteins increased in the TR population. Tellurite resistance protein TehB, lipate-protein ligase B LipB, and the imidazole glycerol phosphate synthase subunit HisF may promote intracellular survival and adaptation of NTHI. TehB provides *Haemophilus* resistance to oxidation, facilitates heme assimilation, and is required for virulence in a bacteremia model (60). Interestingly, TehB is associated with intracellular survival of Corynebacterium diphtheriae (61) and Yersinia pestis (62). Tellurite is a toxic metalloid found in the environment. Therefore, it is likely that the *Haemophilus* TehB has additional functions in the human host. TehB has been proposed to encode an *S*-adenosylmethionine (SAM)-dependent small-molecule methyltransferase (60). Interestingly, our proteomic screen also identified two other SAM-methyltransferases significantly increased in TR NTHI (QueA and a conserved hypothetical), suggesting that increased methyltransferase activity is important for adaptation to nutrient limitation.

LipB was also significantly increased in the transiently restricted population. LipB is a member of the lipoic acid biosynthetic pathway with the lipoyl synthase LipA (Table 1). Lipoate-dependent metabolism is required for optimal cytosolic replication and virulence of Listeria monocytogenes and Burkholderia pseudomallei (63–65). Host-derived lipoic acid is also important for Chlamydia trachomatis intracellular growth and development (66). Ongoing studies will determine the contribution of lipid modifications to invasion and intracellular survival of NTHI in epithelial cells.

The imidazole glycerol phosphate synthase, HisF, is involved in biosynthesis of the essential amino acid histidine as well as in nitrogen metabolism (67). Synthesis of histidine confers a survival advantage in the middle ear, and the *his* operon is significantly more prevalent in NTHI OM strains (68). Histidine biosynthesis is one of the most energy-depleting metabolic pathways (69), and downregulation of histidine biosynthesis may result in a conservation of energy. Our observation that HisF, and not the other members of the biosynthetic pathway, was increased is consistent with prior observations of a requirement for HisF in intracellular replication of Burkholderia pseudomallei (65).

As iron in mammalian cells is sequestered within ferritin or transferrin, increased production of transferrin binding protein 2 may indicate an advantage for TR NTHI to scavenge the low iron stores within the epithelial cell cytoplasm.

The existence of a viable, protected intracellular population of NTHI promoted by transient heme-iron restriction reveals new opportunities for the design of novel therapeutics for chronic and reoccurring OM. Our data underscore the importance of considering extracellular and intracellular niches of NTHI for optimal therapeutic approaches. We demonstrated that blocking macropinocytosis significantly decreased intracellular survival of NTHI by redirecting internalized bacteria through the endolysosomal pathway for degradation (Fig. 7). Similarly, we have previously shown that inhibition of the actin-related protein complex Arp2/3 prevents NTHI uptake into epithelial cells (70). Therefore, it is tempting to speculate that the use of macropinocytosis or actin inhibitors could prevent NTHI persistence and reduce recurrent episodes of OM. Indeed, our data demonstrate the feasibility of the use of macropinocytosis inhibition as independent or adjunct therapies to reduce the intracellular burden of NTHI. Although our data contribute to the growing body of knowledge for the diverse lifestyles and persistent nature of NTHI, a greater understanding of the bacterial factors contributing to IBC formation will be necessary in order to design specific and targeted therapeutics.

## MATERIALS AND METHODS

### Bacterial strains, cell lines, and media.

NTHI strain 86-028NP is a minimally passaged clinical isolate which has been sequenced and characterized in the chinchilla model of OM (20, 71–73). A green fluorescent protein (GFP) reporter strain of 86-028NP was generated by electroporation of plasmid pGM1.1 as previously described (19, 20). For routine culture, NTHI was grown on chocolate agar plates (Fisher Scientific, Pittsburgh, PA). For routine liquid culture, NTHI was grown in an iron-depleted defined iron source (DIS) medium supplemented with β-NAD (74). Where indicated, DIS was supplemented with 2 µg/ml heme (Millipore Sigma, St. Louis, MO). NTHI was transiently restricted or continuously exposed to heme-iron in 24-hour liquid culture as previously described (20). Briefly, strain 86-028NP or 86-028NP(pGM1.1) was grown overnight at 37°C in a 5% CO_2_ atmosphere on chocolate agar plates. Cells were resuspended in DIS, adjusted to an optical density at 490 nm (OD_490_) of 0.65, and diluted 1:10 into prewarmed DIS medium containing either 0 or 2 µg/ml heme. Cultures were grown for 24 h statically at 37°C and 5% CO_2_ and normalized to an OD_490_ of 0.37 in DIS containing 2 µg/ml heme to generate parallel transiently restricted and continuously exposed cultures, respectively.

Normal human bronchial epithelial (NHBE; Lonza, Allendale, NJ) cells were cultured with bronchial epithelial basal medium (Lonza) supplemented according to manufacturer’s specifications in cell culture-treated flasks or plates (Corning, Corning, NY) at 37°C with 90% humidity and 5% CO_2_. Primary chinchilla middle ear epithelial (CMEE) cells were isolated from adult chinchilla middle ear mucosa and cultured in CMEE growth medium at 37°C with 90% humidity and 5% CO_2_ as previously described (20, 75).

### Ethics statement.

All animal experiments were carried out in strict accordance with the accredited conditions in the guide for the care and use of laboratory animals of the National Institutes of Health. The protocol was approved by the Institutional Animal Care and Use Committee at The Research Institute at Nationwide Children’s Hospital. All experimental procedures were performed under xylazine and ketamine anesthesia, and all efforts were made to minimize suffering.

### Chinchilla model of otitis media.

Healthy adult chinchillas (Chinchilla lanigera) were obtained from Rauscher’s Chinchilla Ranch (LaRue, OH). To assess formation of intracellular bacterial communities (IBCs) within middle ear epithelial cells, three cohorts of five chinchillas were transbullarly challenged with 1,077 CFU of transiently restricted or 1,542 CFU of continuously exposed NTHI in a total volume of 300 µl saline per ear. Two days following middle ear challenge, the animals were sacrificed and the middle ear inferior bullae were removed and fixed in buffered 4% paraformaldehyde (PFA) in Dulbecco’s phosphate-buffered saline (DPBS) for 24 h. Fixed bullae were decalcified in 0.35 M EDTA and 0.1 M Tris (pH 6.95) and embedded in paraffin. For immunohistochemistry, 4-µm sections were deparaffinized in xylene followed by antigen retrieval as previously described (76).

### Immunohistochemistry and fluorescent labeling of IBCs *in vivo*.

To assess the formation of IBCs in the chinchilla middle ear, immunohistochemistry was performed on middle ear sections. Nonspecific antibody binding was reduced by incubation for 10 min with 0.01% sodium borohydride, and the inherent fluorescence of the sample was quenched by incubation for 10 min with CAS-block histochemical reagent (Thermo Fisher Scientific, Waltham, MA). The specimens were then incubated for 30 min in Image-iT FX signal enhancer (Thermo Fisher Scientific) to further prevent nonspecific association of dyes with the tissue. NTHI in middle ear sections was detected with a chinchilla antiserum to NTHI outer membrane proteins (OMPs) diluted 1:25 in CAS-block buffer and incubated overnight at 4°C, as previously described (18). The NTHI-antibody complexes were visualized using protein A conjugated with Alexa Fluor 488 (Thermo Fisher Scientific) diluted 1:100 in DPBS for 1 h at room temperature. Host cell membranes were visualized using wheat germ agglutinin (WGA) conjugated with Alexa Fluor 594 (final concentration, 5 µg/ml; Thermo Fisher Scientific), and DNA was visualized with Hoechst 33342 (Thermo Fisher Scientific). Coverslips were mounted on slides with ProLong Gold antifade reagent (Thermo Fisher Scientific) and imaged with an Axiovert 200M inverted epifluorescence microscope equipped with the ApoTome attachment for improved fluorescence resolution and an AxioCam MRM charge-coupled device (CCD) camera and AxioVision software (Carl Zeiss, Inc., Thornwood, NY). Images were processed in Photoshop (Adobe, San Jose, CA) using the levels function applied to every pixel within the image to enhance visualization of fluorescent structures. For rendered images, 0.6-µm optical sections were acquired at 0.6-µm intervals, all images were stacked, and three-dimensional interpolation was performed in Image J (NIH). Orthogonal imaging to visualize the depth of the intracellular communities was performed in Image J (NIH), and a single optical section is depicted in the figure.

### Coculture of nutritionally conditioned NTHI with epithelial cells *in vitro*.

NHBE cells were seeded on glass coverslips in 24-well cell culture dishes (Corning) at 1.9 × 10^4^ cells per well or in 8-well Nunc chamber slides (Thermo Fisher Scientific) at 8,000 cells per well and grown to 90% confluence. CMEE cells were cultured as previously described (20). Transiently restricted or continuously exposed 86-028NP(pGM1.1) was cocultured with epithelial monolayers at a multiplicity of infection (MOI) of 25 bacteria per cell. At 1 h after infection of NHBE cells, cells were washed once with DPBS prior to the addition of fresh bronchial epithelial growth medium (BEGM) and subsequently incubated for 4 or 24 h depending upon the experiment. For CMEE cells, at 5 h postinfection, cells were washed once with DPBS prior to the addition of fresh CMEE growth medium containing 100 µg/ml gentamicin for 30 min. CMEE cells were washed twice with DPBS prior to the addition of fresh medium for a total incubation period of 24 h. Following incubation, monolayers were washed twice with DPBS and fixed on ice for 1 h with 4% PFA. After fixation, monolayers were washed twice with DPBS and host cell membranes were visualized with WGA conjugated to either Alexa Fluor 594 or Alexa Fluor 350 (final concentration, 5 µg/ml) dependent upon other fluorophores used in each experiment. Coverslips were mounted on slides using ProLong Gold antifade reagent. Images were acquired and processed as described above.

### Gentamicin protection assays.

Quantitative gentamicin protection assays were adapted for use with NTHI from an established protocol (77). NHBE cells were seeded at 1.9 × 10^4^ cells per well and grown to 90% confluence in 24-well cell culture plates (Corning). Cells were cultured with transiently restricted or continuously exposed NTHI at a multiplicity of infection (MOI) of 25 per cell for 1 h. To determine the total number of bacteria associated with the cells (intracellular and extracellular), monolayers were lysed with 0.05% saponin for 5 min at room temperature, scraped for 10 s with mini-cell scrapers, and collected in microcentrifuge tubes. The concentration of saponin and duration of cell scraping needed to sufficiently lyse the cells were determined empirically and confirmed by microscopy. The concentration of saponin did not affect the viability of NTHI. To determine the total number of intracellular bacteria, NHBE monolayers were treated with 100 µg/ml gentamicin in BEGM for 30 min to eradicate extracellular bacteria. The concentration and duration of gentamicin treatment sufficient to kill extracellular bacteria were determined empirically. Following gentamicin treatment, monolayers were lysed with 0.05% saponin and scraped with mini-cell scrapers for 10 s per well, and samples were collected in microcentrifuge tubes. All samples were serially diluted and plated to enumerate viable CFU. The percentage of viable bacteria remaining from the original inoculum following gentamicin treatment was calculated by dividing the number of viable bacteria recovered from each well by the number of viable bacteria originally inoculated into each well and multiplying by 100. An invasion index was determined by dividing the percentage of viable intracellular bacteria (gentamicin treated) by the percentage of total intracellular and extracellular bacteria (no gentamicin present). Data from duplicate wells were averaged for each condition and time point, and the experiment was performed in biological triplicate. Statistical significance was determined by Student’s *t* test, *P*  < 0.05 (GraphPad Prism; La Jolla, CA).

### Intracellular survival assays.

To determine the ability of the transiently restricted or continuously exposed NTHI populations to survive within epithelial cells *in vitro*, an intracellular survival assay was performed as follows. Cells were cocultured with transiently restricted or continuously exposed NTHI at an MOI of 25 per cell for 90 min, washed twice with DPBS, and treated with 100 µg/ml gentamicin for 30 min at 37°C and 5% CO_2_ to eradicate extracellular bacteria. Monolayers were washed twice with DPBS and subjected to a second incubation period in antibiotic-free medium for the time indicated in each experiment. Monolayers were treated a second time with 100 µg/ml gentamicin for 30 min at 37°C and 5% CO_2_, washed twice with DPBS, lysed with 0.05% saponin for 5 min at room temperature, and scraped for 10 s with mini-cell scrapers. Samples were collected in microcentrifuge tubes, serially diluted, and plated on chocolate agar to enumerate viable intracellular bacteria. The percentage of viable intracellular bacteria was calculated relative to the inoculum. Data from duplicate wells were averaged for each condition and time point. Eight biological replicates were performed for the 2-h time point, and five biological replicates were performed for the 24-h time point. Significance at each time point was determined by Student’s *t* test, *P*  < 0.05 (GraphPad Prism).

### Pharmacological inhibition of endocytic pathways.

To determine mechanisms of uptake of continuously exposed and transiently restricted NTHI into host epithelial cells, pharmacological compounds were used to systematically inhibit endocytic pathways. Noncytotoxic concentrations of each inhibitor and vehicle were established through 3-(4,5-dimethylthiazol-2-yl)-2,5-diphenyltetrazolium bromide (MTT) reduction assays of uninfected cells using a cytotoxicity detection kit (Promega, Madison, WI). The following pharmacological compounds were used to inhibit endocytosis: 5-(*N*-ethyl-*N*-isopropyl)-amiloride (EIPA; Cayman Chemical, Ann Arbor, MI) was reconstituted in dimethyl sulfoxide (DMSO) to generate a 10-mg/ml working stock and used at a working concentration of 60 µM in cell culture medium to inhibit macropinocytosis; chlorpromazine hydrochloride (Sigma-Aldrich Corp., St. Louis, MO) was resuspended in cell culture medium at a working concentration of 30 µM to inhibit clathrin-mediated endocytosis; cytochalasin D from Zygosporium mansonii (Sigma-Aldrich) was reconstituted in DMSO to generate a 1-mg/ml stock solution and used at a working concentration of 10 µM in cell medium to inhibit F-actin polymerization; methyl-β-cyclodextrin (MβCD; Sigma-Aldrich) was used at working concentrations of 5 mM and 1 mM in cell medium to extract cholesterol and reduce lipid rafts, made fresh in cell culture medium for same-day use. For gentamicin protection assays and fluorescence microscopy, inhibitor concentrations remained constant for pretreatment and the infection period, except for MβCD, which was used to pretreat cells at a concentration of 5 mM and then reduced to 1 mM during the infection period to prevent cytotoxicity. Cells were pretreated with EIPA or MβCD for 2 h at 37°C and 5% CO_2,_ while pretreatment with cytochalasin D or chlorpromazine was for 1 h at 37°C and 5% CO_2_. These inhibitor concentrations and treatment times were determined empirically using fluorescent control cargo conjugates as described below.

### Fluorescence microscopy of control cargo conjugates upon pharmacological inhibition.

Experimental conditions for pharmacological inhibition were determined through optimal inhibition of the following fluorescent control cargo conjugates: cholera toxin subunit B-Alexa Fluor 488 conjugate (Thermo Fisher Scientific; 2-µg/ml final concentration), transferrin-Alexa Fluor 488 conjugate (Thermo Fisher Scientific; 50-µg/ml final concentration), and 70,000-MW dextran-Alexa Fluor 488 conjugate (Thermo Fisher Scientific; 50-µg/ml final concentration). NHBE cells were grown to 90% confluence on coverslips and were pretreated with cell medium with or without inhibitor followed by the addition of cargo conjugates and incubation for 1 h (transferrin and dextran) at 37°C or 20 min on ice followed by 1 h at 37°C (cholera toxin subunit B). Cells were washed twice with DPBS and fixed with 4% PFA overnight at 4°C. PFA was removed, and cells were washed twice with DPBS before coverslips were mounted on slides using ProLong Gold antifade reagent. Cells were imaged and processed as described above. Cytochalasin D reduces cellular F-actin polymerization (76), and pretreatment time and activity of cytochalasin D were monitored by selectively staining for F-actin polymers. Cells were pretreated with the inhibitor for 1 h at 37°C and 5% CO_2_, washed twice with DPBS, and fixed in 4% PFA overnight at 4°C. Following fixation, cells were washed twice with DPBS and permeabilized with 0.1% Triton X-100 in DPBS for 10 min. F-actin was then stained using Alexa Fluor 350-phalloidin according to the manufacturer’s protocol (Thermo Fisher Scientific). Cells were preincubated with Image-iT FX signal enhancer (Thermo Fisher Scientific) for 30 min. A 6.6 µM stock solution of Alexa Fluor 350 was prepared by reconstitution in methanol. A working solution was prepared by diluting 5 µl of the methanolic stock solution into 200 µl of DPBS per coverslip to be stained. Cells were incubated with Alexa Fluor 350-phalloidin for 20 min at room temperature and then washed twice with DPBS and visualized on an Axiovert 200M inverted epifluorescence microscope.

### Fluorescence microscopy following infection of cells treated with pharmacological inhibitors.

To determine the effect of pharmacological inhibition on internalization of nutritionally conditioned NTHI, NHBE cells were seeded at 1.9 × 10^4^ cells per well and grown to 90% confluence on glass coverslips in 24-well plates. Monolayers were pretreated with each inhibitor before and during incubation with transiently restricted or continuously exposed NTHI strain 86-028NP(pGM1.1) at an MOI of 25 per cell for the time points described for each experiment. Following infection, cells were washed twice with DPBS and fixed with 4% PFA overnight at 4°C. Cells were then washed twice with DPBS and incubated with WGA conjugated to Alexa Fluor 594 (final concentration of 5 µg/ml in DPBS) for 10 min at room temperature. Coverslips were washed twice with DPBS and mounted on glass microscope slides using ProLong Gold antifade reagent. Images were acquired and processed as described above. Experiments were performed in biological triplicate, and representative images are shown.

### Gentamicin protection and intracellular survival assays with pharmacological inhibitors.

The gentamicin protection and intracellular survival assay protocols were modified to include a period of pretreatment with cell culture medium containing the inhibitor, as indicated, followed by coculture with transiently restricted or continuously exposed NTHI as described above.

### Immunolabeling of endolysosomal markers.

To examine colocalization of NTHI with endolysosomal markers, NHBE monolayers were seeded at 8,000 cells per well and grown to 90% confluence in 8-well chamber slides. Cells were infected for a total of 4 or 24 h with transiently restricted or continuously exposed NTHI strain 86-028NP(pGM1.1) at an MOI of 25 per cell. Monolayers were washed twice with DPBS and fixed overnight at 4°C in 4% PFA. Cells were then washed twice with DPBS, permeabilized in 1% Triton X-100 in DPBS for 10 min, and blocked with CAS-block for 30 min. Host cell membranes were visualized using WGA conjugated to either Alexa Fluor 350 or Alexa Fluor 594 (final concentration of 5 µg/ml), and early endosomes and lysosomes were visualized by incubation with rabbit anti-early endosomal antigen 1 (EEA1; 1:1,000; Abcam, Eugene, OR) or rat anti-LAMP1 (1:500; Developmental Hybridoma Studies Bank, Iowa City, IA) for 1 h at room temperature, respectively. Cells were washed twice with DPBS and incubated with donkey anti-rabbit IgG conjugated to Alexa Fluor 594 (1:500; Thermo Fisher Scientific) for EEA1 or donkey anti-rat Alexa Fluor 594 (1:500; Thermo Fisher Scientific) for LAMP1 for 1 h at room temperature. Following immunolabeling, cells were washed twice with DPBS and coverslips were mounted on slides using SlowFade Diamond antifade mounting reagent. All images were acquired and processed as described above. Experiments were performed in triplicate, and representative images are shown. Quantification of the number of colocalization events per cell was performed by visually counting 200 total individual cells from three independent experiments for each condition. Statistical significance was determined by a two-tailed Mann-Whitney U test (GraphPad prism).

### Quantification of LAMP1 staining patterns.

NHBE cells were cocultured with either transiently restricted or continuously exposed NTHI strain 86-028NP(pGM1.1) for 5 h and immunolabeled with rabbit anti-LAMP1 (Abcam; 1:500) and donkey anti-rabbit IgG conjugated to Alexa Fluor 594 (1:500; Thermo Fisher Scientific) to visualize LAMP1. Immunofluorescence staining patterns of LAMP1 were visually categorized as diffuse, punctate, or a combination. Quantification of LAMP1 staining patterns was completed by visual counting of ∼100 individual cells by two independent investigators.

### Transmission electron microscopy.

NHBE cells were seeded and grown to 80% confluence in 8-well chamber slides and inoculated with nutritionally conditioned NTHI at an MOI of 25 per cell. At 90 min postinfection, monolayers were washed with DPBS, cell medium was replaced, and infection was allowed to progress for a total of 4 or 24 h as indicated. Monolayers were washed with DPBS and incubated with trypsin (Corning) for 5 min at 37°C and 5% CO_2_, and trypsin was inactivated with trypsin-neutralizing solution (TNS; Lonza). Samples were collected in 15-ml conical tubes and pelleted by centrifugation at 500 × *g* for 10 min. Cell pellets were washed by resuspension in DPBS, followed by centrifugation at 1,000 × *g* for 10 min. Pellets were then fixed in 4% PFA-0.05% glutaraldehyde in DPBS for 1 h on ice. Pellets were stored in cold PBS at 4°C. For immunolocalization of EEA1 and LAMP1, samples were embedded in 10% gelatin and infiltrated overnight with 2.3 M sucrose-20% polyvinylpyrrolidone at 4°C. Samples were trimmed, frozen in liquid nitrogen, and sectioned with a Leica Ultracut UCT7 cryoultramicrotome (Leica Microsystems Inc., Bannockburn, IL). Fifty-nanometer sections were blocked with 5% fetal bovine serum-5% normal goat serum for 30 min and subsequently incubated with rabbit anti-EEA1 antibody (1:500; Abcam) or rabbit anti-LAMP1 (1:200; Abcam) for 1 h followed by secondary anti-rabbit IgG antibody conjugated to 18-nm colloidal gold (1:30; Jackson ImmunoResearch Laboratories, Inc., West Grove, PA) for 1 h. Sections were stained with 0.3% uranyl acetate-2% methylcellulose and viewed on a JEOL 1200 EX transmission electron microscope (JEOL USA Inc., Peabody, MA) equipped with an AMT 8-megapixel digital camera and AMT Image Capture Engine V602 software (Advanced Microscopy Techniques, Woburn, MA). All labeling experiments were conducted in parallel with controls omitting the primary antibody. These controls were consistently negative at the concentration of colloidal gold-conjugated secondary antibodies used in these studies.

### Proteomic analysis of transiently restricted and continuously exposed NTHI. (i) Sample preparation and protein isolation.

NTHI strain 86-028NP(pGM1.1) was continuously exposed to or restricted of heme-iron for 24 h as described under “Bacterial strains, cell lines, and media.” Cultures were normalized to an OD_490_ of 0.37, and 5 ml of each culture was centrifuged at 4,000 × *g* for 15 min at 4°C. Pellets were resuspended in 1 ml 50 mM ammonium bicarbonate buffer (pH 8) and lysed in a high-pressure cell (20,000 lb/in^2^; One Shot model; Constant Systems Ltd., Kennesaw, GA). Samples were centrifuged at 20,000 × *g* at 4°C for 15 min, and the supernatants were stored at −80°C prior to analyses. After thawing, the samples were vortexed and centrifuged at 15,000 × *g* at 4°C. The soluble fractions were lyophilized overnight to dryness and resuspended in 50 µl of 0.2% acid-labile surfactant (ALS-1). Protein concentrations of the supernatants were determined by mini-Bradford assay (Bio-Rad Laboratories, Inc., Hercules, CA). Samples with concentrations of ≥0.3 µg/µl (*n* = 3 per condition) were chosen for further sample processing and proteomic analysis. Ten micrograms of protein per sample was normalized to 0.2 µg/µl in 0.2% ALS-1 and then denatured at 40°C for 10 min. Samples were then reduced with dithiothreitol at a final concentration of 10 mM for 15 min at 80°C, followed by alkylation with a 20 mM final concentration of iodoacetamide at room temperature for 30 min. Samples were cooled to room temperature and then digested with 1 µg sequencing-grade trypsin (Promega) overnight at 37°C. Digestion was quenched, and acid-labile detergent was cleaved by addition of 7 µl of 10:20:70 (vol/vol/vol) trifluoroacetic acid-acetonitrile-water and incubation at 60°C for 2 h. After cooling to 4°C, samples were centrifuged at 15,000 × *g* for 2 min, and the supernatant was transferred into total recovery vials (Waters, Milford, MA). A study pool quality control (SPQC) sample was made by mixing 5 µl from each sample into a separate vial.

### (ii) Quantitative sample analysis.

Each sample was analyzed on a “nanoflow” liquid chromatograph-tandem mass spectrometer (LC-MS/MS) using a Waters nanoAcquity LC interfaced to a Thermo Q Exactive HF with a nanoelectrospray ionization source with tune parameters: spray voltage of 1.8 kV and capillary temperature of 250°C. For quantitative analysis, 3.5 µl (450-ng protein equivalent) was analyzed per injection. A single-pump trapping configuration was used for LC separation, including trapping on an 180-µm by 20-mm Symmetry C_18_ 5-μm column (Waters) and analytical separation on a 75-µm by 250-mm 1.8-µm-particle HSS T3 column (Waters). Trapping utilized 5 µl/min at 99.9/0.1 (vol/vol) water-acetonitrile for 5 min, while analytical separation used a gradient from 5 to 30% acetonitrile (0.1% formic acid) over 90 min at 0.4 µl/min and a column temperature of 55°C.

Label-free quantitative LC-MS/MS on the Q Exactive HF utilized an MS^1^ scan from *m/z* 375 to 1,600 at 120,000 resolution, an automatic gain control (AGC) target of 3e6 ions, and a maximum injection time (IT) of 50 ms. The MS^1^ scan was followed by MS/MS (MS^2^) of the top 12 most abundant ions at 1.2-*m/z* isolation width, 30,000 resolution, AGC target of 1e4 ions, normalized collision energy (NCE) of 27 V, and dynamic exclusion of 20 s.

The SPQC sample was used to condition the column prior to beginning the analysis of the sample set and also before, during, and after the samples for quality control purposes. The run order was an interwoven block design. Data analysis was performed in Rosetta Elucidator v4.0 (Rosetta Biosoftware, Inc., Seattle, WA) feature detection and accurate mass and retention time alignment. Relative peptide abundance was calculated based on area under the curve (AUC) of aligned features across all the runs. MS/MS (*.mgf) data from each run were searched against a custom-built protein sequence database containing a deduplicated aggregate of H. influenzae species, taxonomy downloaded from pubmed.gov (NCBI RefSeq entries) and UniProt (www.uniprot.org), and contaminants from the cRAPome (http://crapome.org/). The database also contained a reversed-sequence “decoy” database for false-discovery rate (FDR) determination. Mascot v2.4 (Matrix Science, Inc., Boston, MA) was used to search data dependent acquisition (DDA) data. Amino acid modifications allowed in database searching included fixed carbamidomethyl Cys (+57) and variable deamidation of Asn and Gln (+1) and oxidation of Met (+16). Tryptic enzyme cleavage rules were followed with up to 2 missed cleavages, a peptide tolerance of ±5 ppm, and a product tolerance of ±0.02 Da. Data were processed to the PeptideTeller data curation algorithm to determine FDR and were annotated at 0.5% peptide FDR. A total of 6,973 peptides and 1,183 proteins were quantified. Peptide intensities were robust mean normalized across all samples, and relative protein abundance was calculated as the simple sum of the peptide intensities to all samples. Peptides were annotated to the single most likely protein under the principles of Occam’s Razor using the ProteinProphet algorithm (78). A summary of the proteomics data is contained in Table 1 (sample identifier information). The percent coefficient of variation (%CV) for the technical replicates analyzed throughout the study showed excellent analytical reproducibility, with a median 4.9% including all proteins, or 4.0% including proteins with 2 or more peptides. Differential expression determination was based on calculation of the fold change (positive reflecting upward in the direction of significance [S], negative reflecting upward in the direction of nonsignificance [NS]), as well as a *t* test after log_2_ transformation of protein intensity values. Student’s *t* test values reported in Table 1 are not corrected for multiple hypotheses.

### Statistical analyses.

Statistical significance was determined with a two-tailed Student *t* test or Mann-Whitney U test as indicated in the figure legends (Graph Pad Prism; La Jolla, CA).

### Accession number(s).

Raw proteomics data for this experiment have been made available on the MassIVE data repository at ftp://massive.ucsd.edu/MSV000082399.

10.1128/mSphere.00286-18.1 TEXT S1 Supplemental materials and methods. Download Text S1, DOCX file, 0.01 MB. Copyright © 2018 Hardison et al. 2018 Hardison et al. This content is distributed under the terms of the Creative Commons Attribution 4.0 International license.

10.1128/mSphere.00286-18.6 FIG S5 Principal-component analysis (PCA) of TR versus CE proteins. A principal-component analysis was utilized as a data quality control for proteomics experiments. Protein intensity (sums of the peptides annotated for each protein) values were log_2_ transformed and z-scaled prior to PCA analysis by Rosetta Elucidator. Samples were labeled according to sample group. The all-protein PCA shows separation by group along the PC 2/3 axis and tight clustering of the study pool quality control (SPQC). Download FIG S5, EPS file, 0.6 MB. Copyright © 2018 Hardison et al. 2018 Hardison et al. This content is distributed under the terms of the Creative Commons Attribution 4.0 International license.
