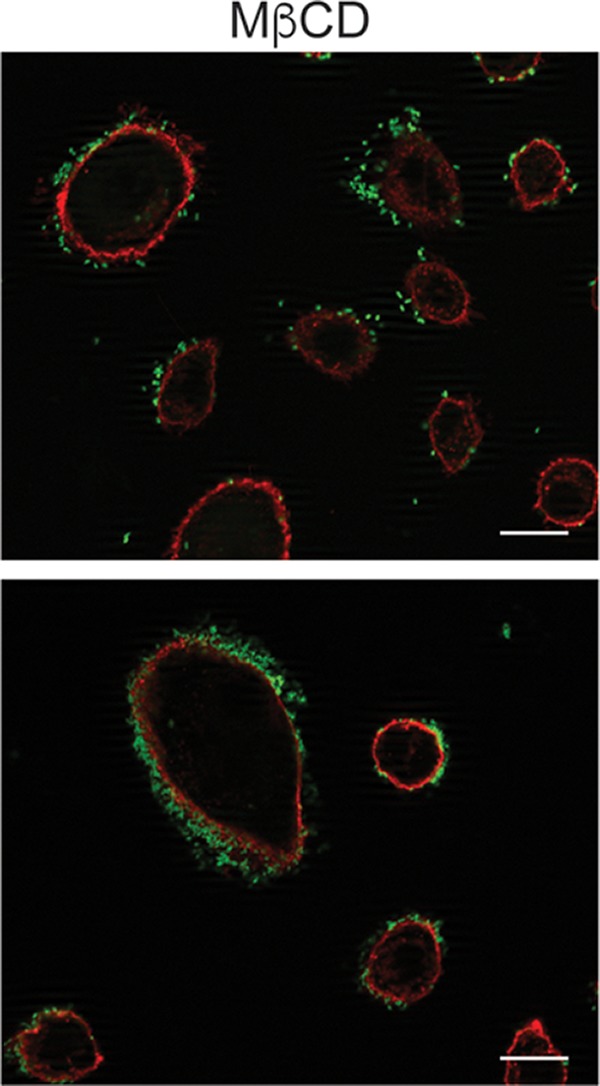# Erratum for Hardison et al., “Transient Nutrient Deprivation Promotes Macropinocytosis-Dependent Intracellular Bacterial Community Development”

**DOI:** 10.1128/mSphere.00562-18

**Published:** 2018-10-31

**Authors:** Rachael L. Hardison, Derek R. Heimlich, Alistair Harrison, Wandy L. Beatty, Sarah Rains, M. Arthur Moseley, J. Will Thompson, Sheryl S. Justice, Kevin M. Mason

**Affiliations:** a Center for Microbial Pathogenesis, The Research Institute at Nationwide Children’s Hospital, Columbus, Ohio, USA; b The Ohio State University College of Medicine, Columbus, Ohio, USA; c Department of Molecular Microbiology, Washington University School of Medicine, St. Louis, Missouri, USA; d Duke Proteomics and Metabolomics Core Facility, Duke Center for Genomic and Computational Biology, Duke University, Durham, North Carolina, USA; e Department of Pediatrics, The Ohio State University, Columbus, Ohio, USA

## ERRATUM

Volume 3, no. 5, e00286-18, 2018, https://doi.org/10.1128/mSphere.00286-18. In Fig. 5B, the two images under “MβCD” were inadvertently duplicated. The correct images are shown below.

**Figure 1:**