# Supplementary figures and images for: Transient Nutrient Deprivation Promotes Macropinocytosis-Dependent Intracellular Bacterial Community Development
Source: mSphere. 2018 Sep 12;3(5):e00286-18. doi: 10.1128/mSphere.00286-18 (PMC6135960; doi:10.1128/mSphere.00286-18)

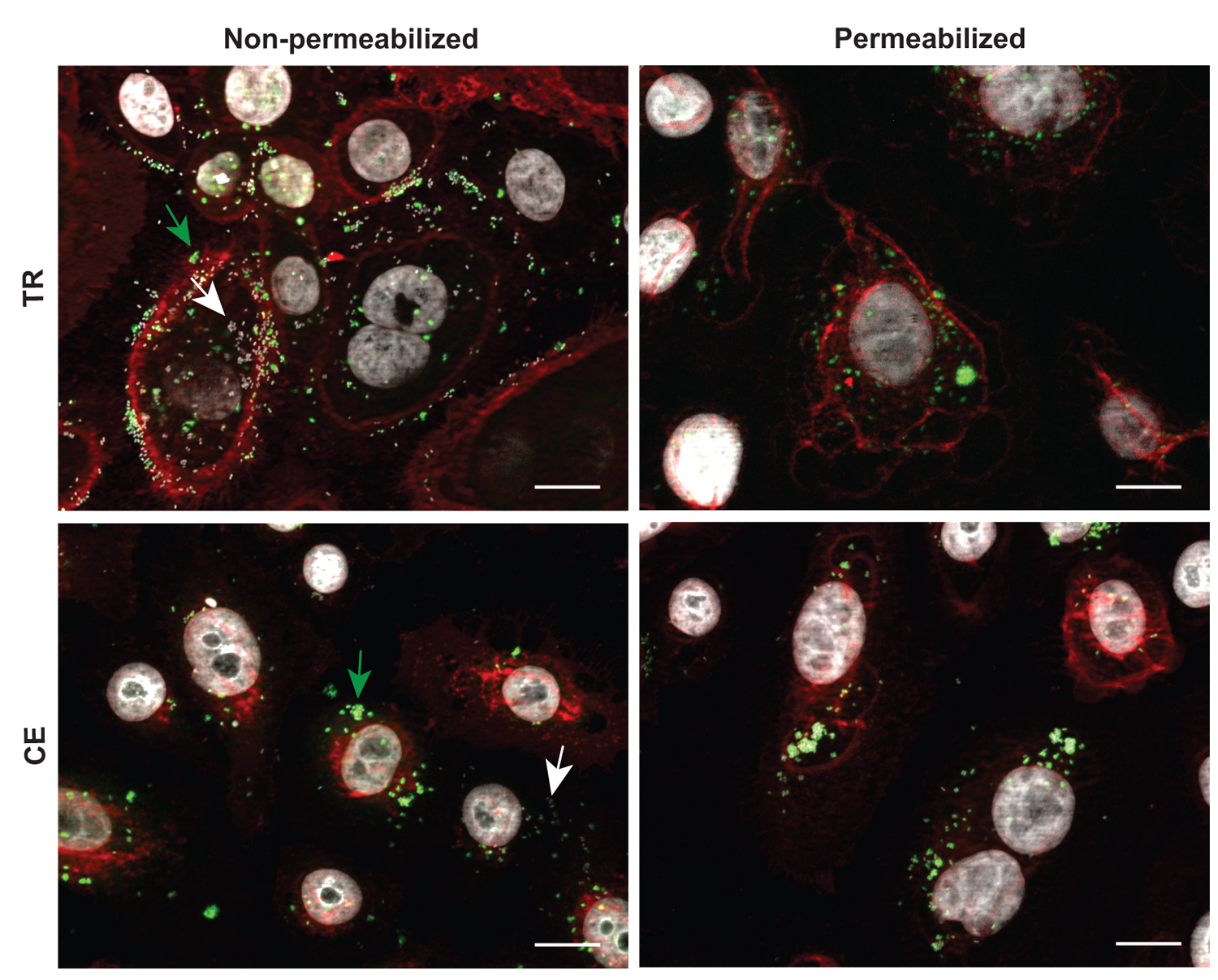

Supplement: FIG S3 [file sph005182638sf3.tif]
