# Supplementary material for: Transient Nutrient Deprivation Promotes Macropinocytosis-Dependent Intracellular Bacterial Community Development
Source: mSphere. 2018 Sep 12;3(5):e00286-18. doi: 10.1128/mSphere.00286-18 (PMC6135960; doi:10.1128/mSphere.00286-18)
Supplement: TABLE S1 [file sph005182638st1.pdf]

Table S1. Pharmacological compounds used to inhibit endocytosis in this study.

Compound	Abbreviation	Target	Mechanism of Action	Positive Control	Reference
Cytochalasin D	CytoD	F-actin	Inhibits actin polymerization	Phalloidin	(1)
Chlorpromazine	CPZ	Clathrin/Receptor-Mediated Endocytosis	Dynamin and/or AP-2 recruitment to clathrin coated vesicles	Transferrin	(2)
Methyl-β-cyclodextrin	MβCD	Lipid rafts	Extraction of cholesterol from cell membrane	Cholera Toxin subunit B	(3)
5-(N-ethyl-N-isopropyl) amiloride	EIPA	Macropinocytosis	Inhibits Na+/H+ exchangers	70,000 MW Dextran	(4)

1. Goddette DW, Frieden C. 1986. Actin polymerization. The mechanism of action of cytochalasin D. J Biol Chem 261:15974-80.
2. Daniel JA, Chau N, Abdel-Hamid MK, Hu L, von Kleist L, Whiting A, Krishnan S, Maamary P, Joseph SR, Simpson F, Haucke V, McCluskey A, Robinson PJ. 2015. Phenothiazine-derived antipsychotic drugs inhibit dynamin and clathrin-mediated endocytosis. Traffic 16:635-54.
3. Lopez CA, de Vries AH, Marrink SJ. 2011. Molecular mechanism of cyclodextrin mediated cholesterol extraction. PLoS Comput Biol 7:e1002020.
4. Koivusalo M, Welch C, Hayashi H, Scott CC, Kim M, Alexander T, Touret N, Hahn KM, Grinstein S. 2010. Amiloride inhibits macropinocytosis by lowering submembranous pH and preventing Rac1 and Cdc42 signaling. J Cell Biol 188:547-63.
