# Supplementary material for: Transient Nutrient Deprivation Promotes Macropinocytosis-Dependent Intracellular Bacterial Community Development
Source: mSphere. 2018 Sep 12;3(5):e00286-18. doi: 10.1128/mSphere.00286-18 (PMC6135960; doi:10.1128/mSphere.00286-18)
Supplement: TEXT S1 [file sph005182638s1.docx]

**SUPPLEMENTAL MATERIAL**

***Supplemental Methods***

**Visual localization of intra- vs extracellular NTHI**

To confirm the subcellular localization of NTHI, NHBE monolayers were co-cultured with continuously exposed or transiently restricted NTHI strain 86-028NP in an 8-well chamber slide (as described in the Materials and Methods) for 4 hours and fixed with 4% PFA. Cells were then either permeabilized with 0.1% Triton X-100 for 10 minutes at room temperature or non-permeabilized. NTHI were labeled with anti-OMP antiserum diluted 1:25 in CAS-Block for 3 hours at room temperature. Cells were washed twice with DPBS and NTHI was detected with protein A-Alexa Fluor 488 diluted 1:500 in DPBS for 1 hour at room temperature. Cells were then washed twice with DPBS and coverslips were mounted on slides using ProLong Gold with DAPI (Thermo Fisher Scientific) to visualize host cell and bacterial nuclei, pseudocolored white. In permeabilized cells, both intracellular and extracellular NTHI are observed as green/white. In non-permeabilized cells, intracellular NTHI are observed as white and extracellular NTHI are observed as green. All images were taken with an Axiovert 200M inverted epifluorescence microscope. Experiments were performed in triplicate and representative images are shown.

**Cytotoxicity Assay**

To determine if nutritionally conditioned NTHI was cytotoxic to mammalian cells at the time points used in this study, NHBE cells were seeded into 96 well cell culture plates at 1.9e^4^ cells per well and allowed to grow to 80-90% confluency. Transiently restricted or continuously exposed NTHI strain 86-028NP were co-cultured with the confluent NHBEs at an MOI of 25 per cell for 4 or 24 hours. Cytotoxic effect of nutritionally conditioned NTHI was determined by measuring LDH release using the CytoTox 96 Non-Radioactive Cytotoxicity Assay (Promega) according to the manufacturer’s protocol.

**Principal components analysis**

A principal components analysis (PCA) was utilized as a data quality control for proteomics experiments. Protein intensity (sums of the peptides annotated for each protein) values were log2 transformed and z-scaled prior to PCA analysis by Rosetta Elucidator. Samples were labeled according to sample group (transiently restricted, continuously exposed, or Study Pool Quality Control).
